# Study on Optimal Nitrogen Application for Different Oat Varieties in Dryland Regions of the Loess Plateau

**DOI:** 10.3390/plants13212956

**Published:** 2024-10-22

**Authors:** Yuejing Qiao, Luming Zhao, Duo Gao, Lijing Zhang, Laichun Guo, Junyong Ge, Yaqi Fan, Yiyu Wang, Zhixia Yan

**Affiliations:** 1 College of Agriculture, Shanxi Agricultural University, Jinzhong 030801, China; qyjsxau@126.com (Y.Q.); zhao736317865@163.com (L.Z.); gaoduos20222188@163.com (D.G.); zlj15808374383@163.com (L.Z.); fanyq323@163.com (Y.F.); wangyiyu0528@163.com (Y.W.); yzx1810220206@163.com (Z.Y.); 2 Baicheng Academy of Agricultural Sciences, Baicheng 137000, China; 3 Zhangjiakou Academy of Agricultural Sciences, Zhangjiakou 075000, China; gejunyong1987@163.com

**Keywords:** oats, yield, quality, nitrogen application, nitrogen fertilizer utilization efficiency

## Abstract

The present study endeavored to tackle the challenges posed by limited diversity in oat varieties and suboptimal nitrogen fertilizer utilization in the arid landscapes of the Loess Plateau. We selected three oat varieties, including early-maturing oats (E), medium-maturing oats (M), and late-maturing oats (L). In 2022, four nitrogen applications were set up as CK (0 kg N ha^−1^), N1 (60 kg N ha^−1^), N2 (90 kg N ha^−1^), and N3 (120 kg N ha^−1^). We introduced two additional nitrogen applications, N4 (180 kg N ha^−1^) and N5 (240 kg N ha^−1^), in 2023. The two-year study results demonstrated a significant increase in oat yield due to nitrogen application (*p* < 0.05). The highest grain yield was observed for E oats at 2216.63 kg·ha^−1^ under the N3 treatment, while M and L oats had the highest grain yields at 2505.43 kg·ha^−1^ and 2946.30 kg·ha^−1^ under N4, respectively. The protein content of L oats reached a peak of 14.15% under N4, and the order of protein contents in oat protein components was globulin > gliadin> glutenin > albumin. The β-glucan content of L oats reached a peak of 4.92% under N3. The nitrogen fertilizer utilization efficiency (NFUE) of the three oats was highest under N2. L oats exhibited enhanced NFUE owing to an elevated pre-flowering nitrogen translocation amount (PrNTA), with a 42.94% and 29.51% increase relative to E and M oats, respectively. The pre-flowering nitrogen translocation contribution (PrNTC) in oats surpassed the post-flowering nitrogen accumulation contribution (PoNAC). Therefore, nitrogen application positively impacted oat growth, yet excessive application had an inhibitory effect. There is a significant positive correlation among oat yield, quality, nitrogen accumulation, and utilization efficiency. In summary, oat crops exhibited optimal performance in terms of yield, quality, and nitrogen use efficiency when nitrogen application rates ranged between 90 and 180 kg·ha^−1^. Late-maturing oats coincide with the rainy and hot season in the northern dryland regions, making them more suitable for planting in the dryland areas of the Loess Plateau.

## 1. Introduction

The Loess Plateau, located in north-central China, represents a typical rainfed agricultural region characterized by its fragile ecological environment. It is crucial to screen suitable crops species and varieties whose water requirements align with the local rainfall patterns. Oat (*Avena nuda* L.), a member of the genus Avena within the Poaceae family [1], is an annual herb and a high-quality cereal crop renowned for its resilience to cold temperatures, drought conditions, and suboptimal soil fertility [2]. Oats are commonly utilized as a source of nutrition for both humans and animals. The seeds are high in nutrients like proteins, fats, and beta-glucans, which can benefit both human and animal health. Additionally, studies have demonstrated that they effectively reduce cholesterol in the bloodstream and mitigate the risk of heart disease [3,4]. Therefore, the cultivation practices and varietal adaptability of oats have been receiving increasing attention. However, the response of different oat varieties to nitrogen fertilizer remains unclear.

The growth of oats is influenced not only by their genetic characteristics, but also by external factors, such as water, nitrogen, and cultivation practices, which have a large impact on oat yield and quality [5]. Wang et al. [6] showed that nitrogen plays a crucial role in determining oat yield and quality. An appropriate nitrogen application amount enhances the vertical gradient of nitrogen distribution within crop crowns and leaves, while also boosting the translocation and contribution rate of nitrogen to grains. This, in turn, elevates the grain nitrogen content and grain quality [7]. Oat varieties have exhibited varying responses to nitrogen [8]. Given that oat varieties with different growth period differ in overall yield potential, it is plausible that optimal nitrogen application rates could vary [9]. Khalid et al. [10] applied a total of 150 kg·ha^−1^ of pure N for oats and obtained the highest N fertilizer utilization. Cui et al. [11] investigated the nitrogen utilization and translocation traits of diverse wheat cultivars at varying nitrogen application levels, and revealed that nitrogen application enhances the above-ground nitrogen accumulation in plants and the efficacy of nitrogen translocation towards seeds prior to anthesis. Ma et al. [12] discovered that nitrogen application substantially promoted the translocation of stored nitrogen to seeds; however, excessive application hindered this process. Additionally, variations in nitrogen translocation were observed among different cultivars. Specifically, the contribution of nitrogen to seeds from pre-flowering translocation surpassed that from post-flowering assimilation, with the latter showing a declining trend. When the nitrogen application continued to increase, the seed protein content remained stable after reaching a maximum level, while the nitrogen concentration in the nutrient organs continued to increase, and the nitrogen translocation efficiency from the nutrient organs to the seed was suppressed at excessively adequate levels of nitrogen supply [13]. Elevated nitrogen inputs promote vigorous growth in nutrient organs, leading to substantial material consumption that reduces the translocation of assimilates and nitrogen to the seed. This heightened vulnerability to plant lodging subsequently limits both the seed yield and quality [14]. Exploring effective nitrogen fertilizer transport methods and identifying nitrogen-efficient oat varieties are crucial in enhancing nitrogen utilization, mitigating nitrogen loss, and addressing environmental concerns such as soil compaction, acidification, and excessive greenhouse gas emissions.

In recent years, there has been a surge in scholarly research focusing on regional adaptive variety comparison tests in introduced crop varieties that exhibit both high yields and superior quality. Most have focused on grain crops such as wheat [15], cereals [16], and sorghum [17], with little research conducted in relation to the varieties of oats. The research on oats primarily centers on production performance [18], nutritional quality [19], and feeding value [20], with inadequate attention given to the adaptability of different oat varieties to regional conditions. Reports of the screening of naked oats with varying growth periods in the northern dryland regions are uncommon in research reports. Therefore, our study on the adaptability of oats with different growth periods in the dry farming area of the Loess Plateau involved three aspects. Firstly, we aimed to determine the optimal nitrogen application rates for oats, considering the delicate balance between yield and quality. Secondly, we sought to enhance nitrogen fertilizer use efficiency, minimize fertilizer inputs, and investigate the potential of nitrogen application for oats across varieties with various growth periods. Thirdly, we aimed to clarify the contribution rate of pre- and post-flowering nitrogen transport to oat grains. Consequently, we would identify the optimal oat variety for dryland regions and establish a theoretical foundation for the introduction of oat varieties into these areas, along with the optimization of nitrogen fertilizer strategies.

## 2. Materials and Methods

### 2.1. Experimental Site

**Figure 1:**
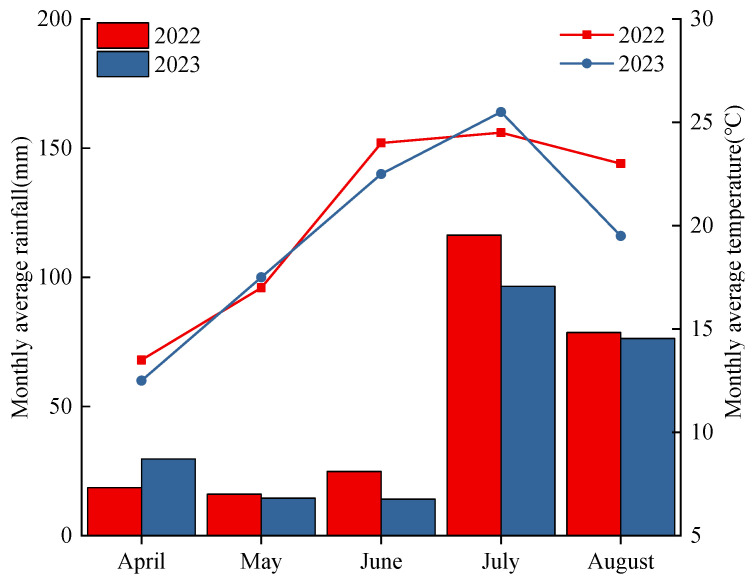
Rainfall and temperature at the experimental site during the oat growing season in 2022 and 2023.

The experiment was conducted from April 2022 to August 2023 at Shenfeng Experimental Base of Shanxi Agriculture University, Taigu District, Jinzhong City, Shanxi Province (37°12′21″ N, 112°28′40″ E). The experimental site has a temperate continental climate, with an elevation of 799.14 m, a total cumulative temperature of 3900–4100 °C, and a frost-free period of 170–175 d. The soil is a brown calcic soil, Figure 1 shows the average temperature and precipitation during the oat growing season, and Table 1 shows the physical and chemical properties of the soil before sowing. The soil organic matter was quantified by the K_2_Cr_2_O_7_-H_2_SO_4_ oxidation procedure. The semi-micro Kjeldahl method and alkalolytic diffusion method were used for total soil nitrogen and alkali hydrolyzed nitrogen, respectively. The soil available phosphorus and available potassium were determined using the molybdenum blue colorimetric method and flame spectrophotometry, respectively. pH was determined using a pH meter following the shaking of the soil/water (1:2.5 *w*/*v*) suspension.

### 2.2. Experimental Design

In this experiment, a split-plot experimental design was used, with the main area being used for the oat maturing varieties Bayou 6 (early-maturing oat/E), Bayou 1 (medium-maturing oat/M), and Bayou 14 (late-maturing oat/L), all of which were supplied by Zhangjiakou Academy of Agricultural Sciences, and the sub-area being used for the nitrogen application treatments. Four nitrogen application treatments were set up in 2022: no nitrogen application (CK) and nitrogen applications of 60 kg·ha^−1^ (N1), 90 kg·ha^−1^ (N2), and 120 kg·ha^−1^ (N3); two additional N application treatments of 180 kg·ha^−1^ (N4) and 240 kg·ha^−1^ (N5) were set up in 2023. Each treatment was repeated three times, with a plot area of 9 m^2^ (3 × 3 m^2^), and 1 m isolation space between plots. The main plots and split plot were randomly distributed. The N, P, and K fertilizers were urea, superphosphate, and potassium chloride, respectively, and the application rate for both P and K fertilizers was 120 kg·ha^−1^; all fertilizers were applied once before sowing, the row spacing was 25 cm, and the seeding rate was 150 kg·ha^−1^. The field was plowed in November, followed by rotary tillage prior to sowing each year. The phenological period of oats showed in Table 2.

### 2.3. Measurement and Methods

#### 2.3.1. Dry Matter Accumulation and Yield of Oats

0.5 m of row was sampled for both the flowering and maturity stages of oats. During the flowering stage, the oat samples were segmented into three components: the stem, leaf, and spike. In contrast, at maturity (full ripening period), the samples were categorized into four parts: the stem, leaf, rachis + glume, and seed. Subsequently, the oat parts were placed in an oven for 0.5 h at 105 °C to terminate their biological activity, followed by drying at 85 °C until a constant weight was achieved. Afterwards, they were weighed, ground, sieved, and stored. Upon reaching maturity, a 1 m^2^ area of unsampled oats was harvested from each plot, and the grains were threshed for yield assessment.

#### 2.3.2. Nitrogen-Related Indicators

Oat samples were digested by the sulfuric acid–hydrogen peroxide method, and the nitrogen content was determined by the indophenol blue colorimetric method. The nitrogen accumulation was calculated based on the dry matter weight of oats for nitrogen-related indexes. Nitrogen utilization- and transport-related indexes were calculated, with references to Sun et al. [21], Cui et al. [22], and Li et al. [23].

Grain nitrogen accumulation (GNA, kg·ha^−1^) = Dry weight of grain × Nitrogen content of grain;

Plant nitrogen accumulation (PNA, kg·ha^−1^) = Dry weight of plants at maturity × Nitrogen content of individual plants at maturity;

Nitrogen fertilizer utilization efficiency (NFUE, %) = (Total nitrogen uptake by plants in the nitrogen applied area − Total nitrogen uptake by plants in the no-nitrogen-applied area)/Amount of nitrogen applied × 100%;

Nitrogen fertilizer productivity (NFP, kg·kg^−1^) = Grain yield in nitrogen application area/Amount of nitrogen applied;

Nitrogen agronomic efficiency (NAE, kg·kg^−1^) = (Grain yield in nitrogen-applied area − Grain yield in no-nitrogen-applied area)/Amount of nitrogen applied;

Pre-flowering nitrogen translocation amount (PrNTA, kg·ha^−1^) = Nitrogen accumulation at flowering − Nitrogen accumulation in nutrient organs at maturity;

Pre-flowering nitrogen translocation contribution rate (PrNTC, %) = Pre-flowering nitrogen translocation amount/Nitrogen accumulation in grain at maturity × 100%; 

Nitrogen translocation rate (NTR, %) = Pre-flowering nitrogen translocation amount/Nitrogen accumulation during flowering × 100%;

Post-flowering nitrogen accumulation amount (PoNAA, kg·ha^−1^) = Grain nitrogen accumulation − Pre-flowering nitrogen translocation amount; 

Post-flowering nitrogen accumulation contribution rate (PoNAC, %) = Post-flowering nitrogen accumulation amount/Grain nitrogen accumulation × 100%.

#### 2.3.3. Grain Protein and β-Glucan Contents of Oats

The protein components were extracted using the continuous extraction method, and they were extracted with distilled water (albumin), 0.5 mol/L NaCl (globulin), 75% ethanol (gliadin), and 0.1 mol/L NaOH (glutenin), respectively. The protein components and protein content of samples were determined using the Kjeldahl method with a protein–nitrogen coefficient of 5.7 [24].

Grain β-glucan content was determined using the Megazyme Mixed Glucan Test Kit, Irish.

### 2.4. Statistical Analysis

Microsoft Excel 2021 was used to process the data, and SPSS 23.0 software was used to perform the analysis of variance, correlation analysis, and principal component analysis and to compare the means of the treatments based on the least significant (LSD) test at the 0.05 level of probability. Multi-way ANOVA was used to evaluate the effects of varieties and nitrogen and interactions on each of the variables; the results of the experiment were averaged over at least three replications and plotted graphically using Origin 2021.

## 3. Results

### 3.1. Effect of Nitrogen Application on Oat Yields

**Figure 2:**
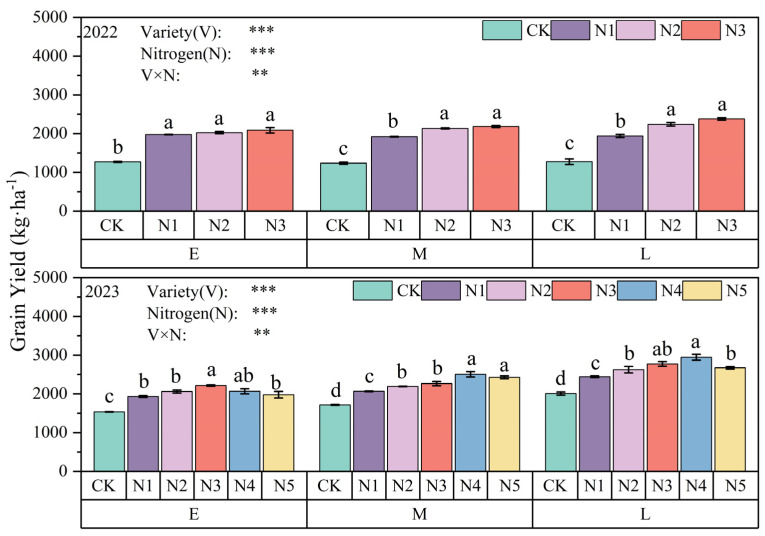
Grain yield of different oat varieties with different nitrogen applications. Note: Different lowercase letters for the same variety indicate that the difference between different nitrogen fertilizer levels of the same variety is significant at the *p* < 0.05 level; * indicates that the difference is significant at the 0.05 level; ** indicates that the difference is significant at the 0.01 level; *** indicates that the difference is significant at the 0.001 level; and ns indicates that the difference is not significant. The same is true for the following Figure 4, Figure 5 and Figure 6.

In 2022, grain yield increased significantly with increasing N application, with 52.04–55.57%, 59.43–75.59%, and 64.18–86.55% increases in yield for the N1, N2, and N3 treatments in E, M, and L oats, respectively, compared to the CK treatment. In 2023, with the addition of two N application treatments, the highest grain yield for E oats (2216.63 kg·ha^−1^) was recorded with the N3 treatment, and the highest grain yields of M oats and L oats were recorded with the N4 treatment (M oats, 2505.43 kg·ha^−1^; L oats, 2946.30 kg·ha^−1^). Under the N4 treatment, the grain yield of L oats increased by 17.59% and 42.46% compared to E oats and M oats. The interaction of maturity and N fertilizer showed significant differences (*p* < 0.01) in grain yield (Figure 2).

**Figure 3:**
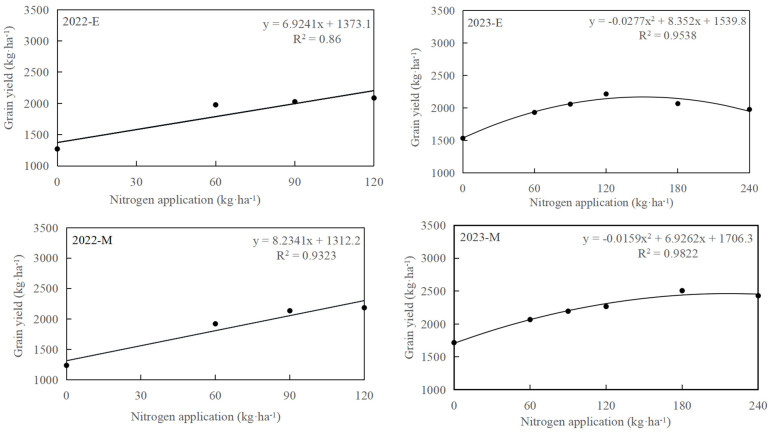

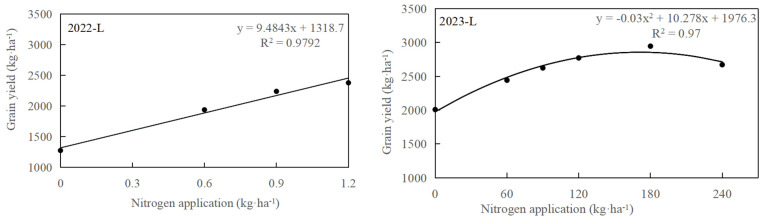
The relationship between the oat grain yield and nitrogen application level.

Regression analysis was conducted on the oat grain yield and nitrogen application rate (Figure 3). In 2022, the grain yield of three oat varieties showed an upward trend with the increase in nitrogen application rate. After adding two nitrogen application treatments in 2023, the grain yield of three varieties showed a quadratic curve relationship with the increase in nitrogen application, and the fitting effect reached a significant level. Applying an optimal level of nitrogen fertilizer effectively enhances oat yield. Nonetheless, exceeding a critical threshold of nitrogen application can lead to a significant reduction in oat grain yield. According to the regression equation between the nitrogen application amount (X) and grain yield (Y), when the nitrogen application is 150.76 kg·ha^−1^, E oats can obtain the highest oat grain yield of 2169.37 kg·ha^−1^. When the nitrogen application is 217.81 kg·ha^−1^, M oats can obtain the highest yield of 2460.58 kg·ha^−1^. Meanwhile, L oats can obtain the highest yield of 2856.61 kg·ha^−1^ at 171.30 kg·ha^−1^ nitrogen application.

### 3.2. Effect of Nitrogen Application on Oat Grain Protein and β-Glucan Content

**Figure 4:**
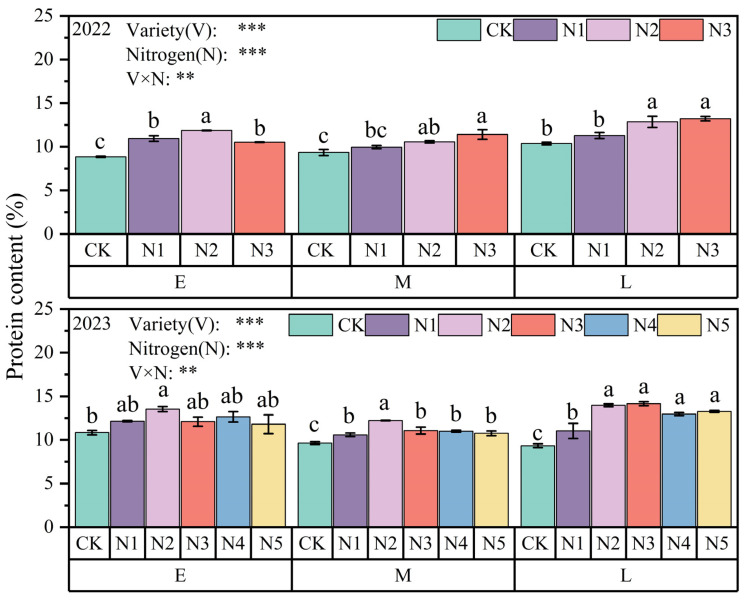
Grain protein content of different oat varieties under different nitrogen applications.

As shown in Figure 4, nitrogen application significantly increased the protein content of oat grain (*p* < 0.05). In the two growing seasons, the grain protein content of E oats increased first and then decreased with the increase in nitrogen application and reached its highest value under the N2 treatment. Similarly, the grain protein content of M oats also performed best under the N2 treatment. The grain protein content of L oats peaked under the N3 treatment, but it is worth noting that the differences between the N2, N3, N4, and N5 treatments were not significant. Specifically, the grain protein content of L oats reached 14.15%, which increased by 4.60% and 15.85% compared with the E and M varieties, respectively.

**Figure 5:**
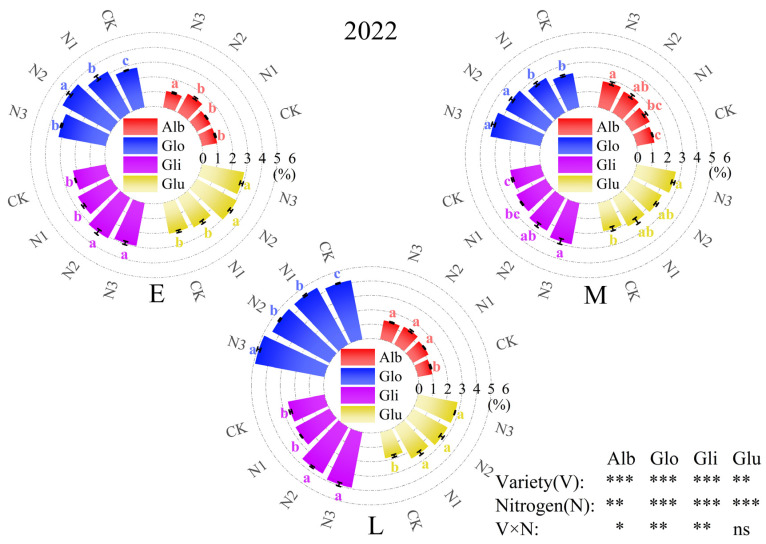

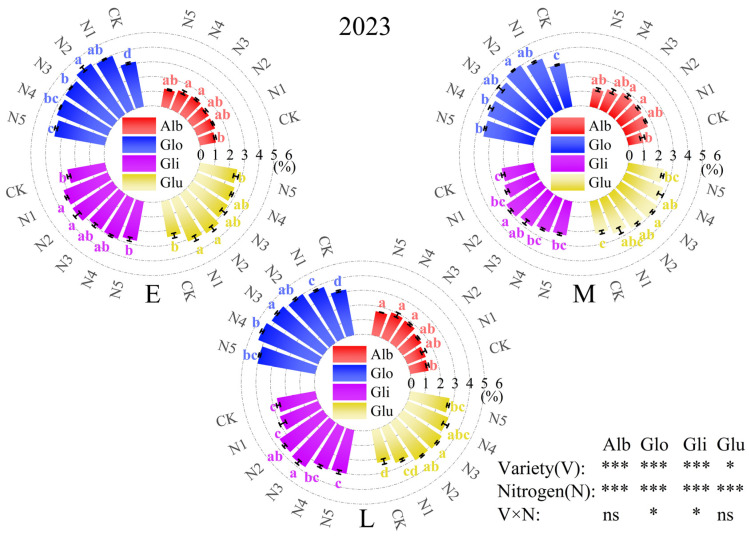
Grain protein component contents of different oat varieties under different nitrogen applications. Note: Alb, albumin; Glo, globulin; Gli, gliadin; and Glu, gluten.

The order of protein component content in the grains of three varieties of oat was as follows: Glo > Gli > Glu > Alb (Figure 5). In comparison among the three varieties, without nitrogen application, the Alb content was highest in M oats, the Glo and Gli content was highest in L oats, and the Glu content was highest in E oats. Nitrogen application significantly increased the content of grain protein components (*p* < 0.05), with Alb reaching the highest value with the N4 treatment in M oats, the Glo and Gli content reaching the highest value with the N4 treatment in L oats, and the Glu content reaching the highest value with the N3 treatment in E oats. The interaction effect of variety and nitrogen fertilization on Alb, Glo, Gli, and total protein content reached a significant level (*p* < 0.05) in 2022. The interaction effect of variety and nitrogen fertilization on Glo, Gli, and total protein content reached a significant level (*p* < 0.05) in 2023.

**Figure 6:**
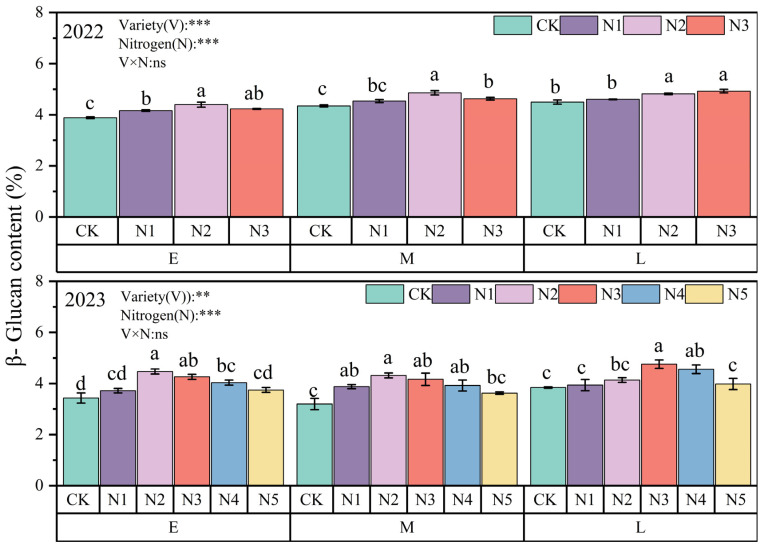
β-glucan content of different oat varieties under different nitrogen applications.

Nitrogen application increased the β-glucan content of oat grains significantly (*p* < 0.05); the β-glucan content of oats showed a tendency of increasing and then decreasing with the increase in nitrogen application (Figure 6). Without nitrogen application, the β-glucan content of L oats was the highest in comparison with other varieties. The β-glucan content of E and M oats reached its peak under the N2 treatment, while that of L oats reached its peak under N3, with a maximum of 4.93%. The β-glucan content of L oats was more sensitive to high amounts of nitrogen fertilizer, and E oats increased more after nitrogen application.

### 3.3. Effect of Nitrogen Application on Dry Matter and Nitrogen Accumulation

**Figure 7:**
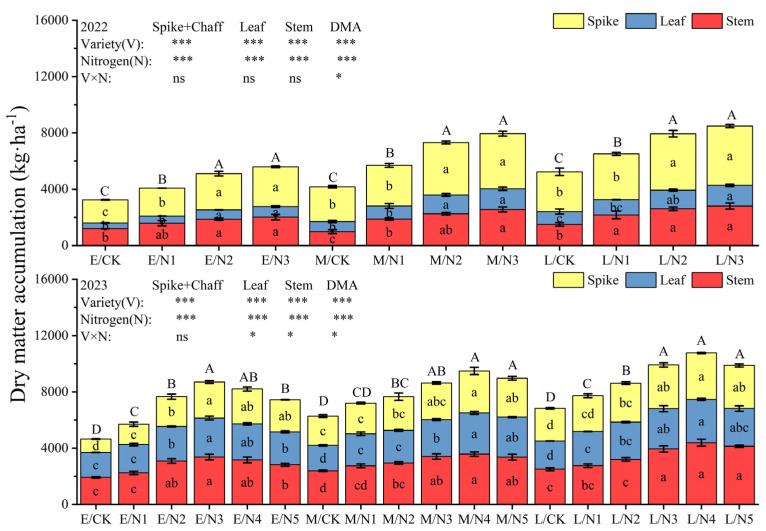
Dry matter accumulation in various organs of oats at the flowering stage. Note: Different capital letters on the columns indicate significant (*p* < 0.05) differences in the dry matter accumulation of the same variety of oats with different nitrogen applications. Different lowercase letters on the columns indicate significant (*p* < 0.05) differences in dry matter accumulation in oat organs of the same variety under different nitrogen application rates. * indicates that the difference is significant at the 0.05 level; ** indicates that the difference is significant at the 0.01 level; *** indicates that the difference is significant at the 0.001 level; and ns indicates that the difference is not significant. The same is true for the Figure 7, Figure 8, Figure 9 and Figure 10.

**Figure 8:**
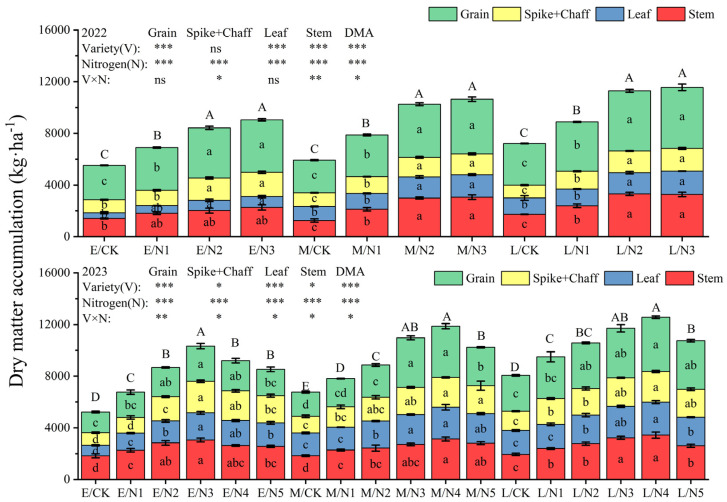
Dry matter accumulation in various organs of oats at maturity stage.

The aboveground dry matter accumulation of oats showed a significant upward trend with the increase in N application and reached its highest at the N3 level in 2022 (Figure 7 and Figure 8). At the flowering stage, the order of dry matter accumulation in each organ under CK was spike > stem > leaf. At maturity, the dry matter accumulation of L oats reached a maximum of 11,561.18 kg·ha^−1^ under N3, which increased by 27.81% and 8.62% compared with that of E and M oats. In 2023, the dry matter accumulation of oats showed an increasing and then decreasing trend with the increase in nitrogen application, and at the maturity stage, with the increase in nitrogen application, the dry matter accumulation of L oats reached its highest value of 12567.08 kg·ha^−1^ in the N4 treatment, which increased by 21.71% and 5.87%, respectively, in comparison with that of E and M oats. The interaction of variety and nitrogen fertilizer had significant (*p* < 0.05) effects on whole-plant dry matter accumulation (DMA) at the flowering stage and on stem, (spike + chaff), and DMA at the mature stage.

**Figure 9:**
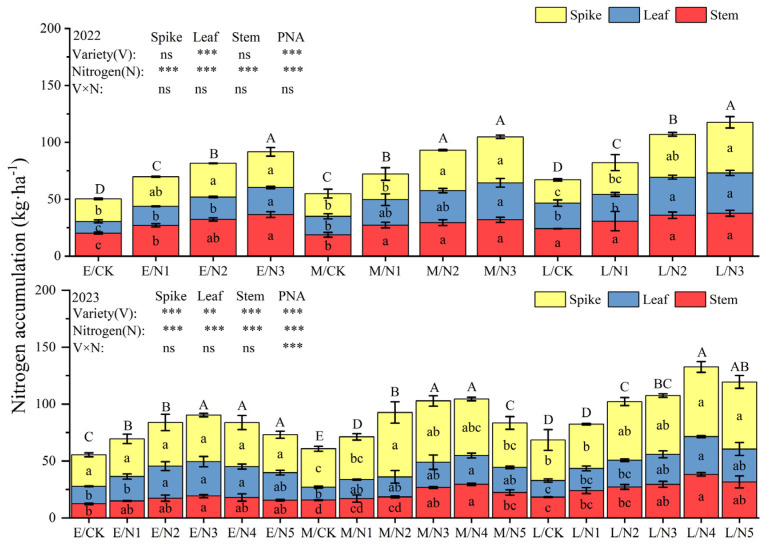
Nitrogen accumulation in various organs of oats at the flowering stage.

**Figure 10:**
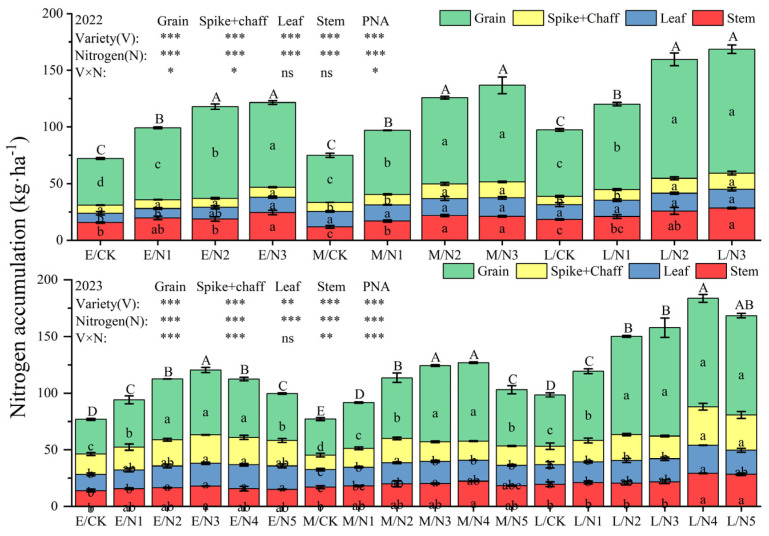
Nitrogen accumulation in various organs of oats at the maturity stage.

During the flowering stage, the application of nitrogen fertilizer increased the nitrogen accumulation in stems, leaves, (spike axis + glume), and grains to varying degrees (Figure 9). The stem and leaf nitrogen accumulation in L oats was the highest (38.29 kg·ha^−1^ and 35.45 kg·ha^−1^), which was significantly higher than that of E and M oats. Nitrogen accumulation in the nutrient organs of oats (stems and leaves) showed a decreasing trend from the flowering stage to the maturity stage, whereas nitrogen accumulation in reproductive organs (spike + chaff and grain) showed an increasing trend. With the elevation in nitrogen application, the nitrogen accumulation of oats in 2022 showed an upward trend, but in 2023, it showed a tendency of increasing and then decreasing, and L oats reached the highest value of 183.68 kg·ha^−1^, which was significantly higher than that of E oats and M oats by 51.14% and 44.63% (Figure 10).

### 3.4. Nitrogen Fertilizer Utilization Efficiency and Nitrogen Translocation Efficiency in Oats

As shown in Table 3, the interaction of variety and nitrogen fertilizer significantly (*p* < 0.05) affected the NFUE, NFP, and NAE of oats. The NFUE of all varieties exhibited a trend of an initial increase followed by a decrease as nitrogen (N) application rates increased; it was highest under N2 and lowest under N5, and the highest NFUE was measured in L oats (69.19%), outperforming E and M oats by an average of 40.03% and 31.92%, respectively, over the two-year study. In terms of NFP and NAE, the three varieties of oats showed a significant decreasing trend (*p* < 0.05) with increasing N application.

As shown in Table 4, the interaction of variety and nitrogen fertilizer significantly affected PrNTA, NTR, and PoNAA (*p* < 0.05 or *p* < 0.001). PrNTA showed a tendency to increase and then decrease with nitrogen application, and the PrNTA values of all nitrogen fertilizer treatments were significantly higher than that of CK (*p* < 0.05), and PrNTA was greater than PoNAA, indicating that pre-flowering nitrogen translocation was the main source of nitrogen in grains. The highest level of PrNTA was observed in E oats under the N3 treatment, whereas in L oats, this was seen under the N4 treatment. E oats reached the highest NTR (64.39%) in 2022.

### 3.5. The Correlation of Oat Characteristics

**Figure 11:**
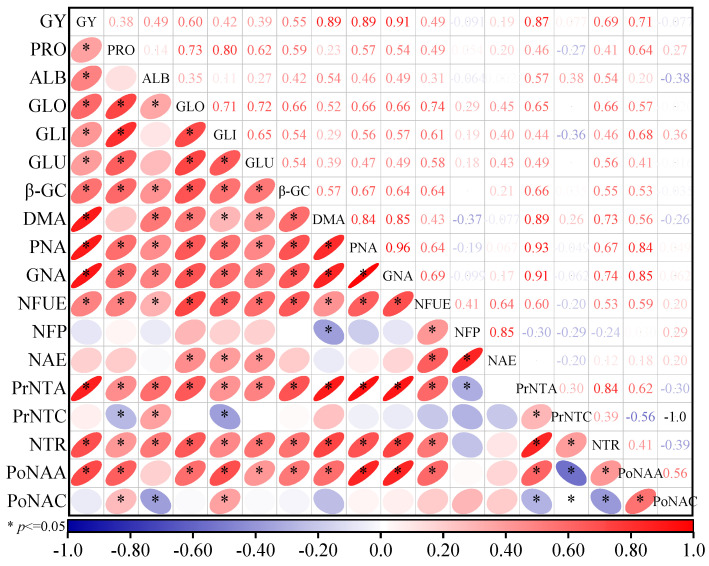
Correlation analysis of various oat characteristics. Note: GY, grain yield; PRO, protein content; ALB, albumin; GLO, globulin; GLI, alcohol-soluble protein; GLU, glutelin; β-GC, β-Glucan; DMA, dry matter accumulation; GNA, grain nitrogen accumulation; PNA, plant nitrogen accumulation; NFUE, nitrogen fertilizer utilization efficiency; NFP, nitrogen fertilizer productivity; NAE, nitrogen fertilizer agricultural efficiency; PrNTA, pre-flowering nitrogen translocation amount; PrNTC, pre-flowering nitrogen translocation contribution; NTR, pre-flowering nitrogen translocation rate; PoNAA, post-flowering nitrogen accumulation amount; PoNAC, post-flowering nitrogen accumulation contribution. Red represents a positive correlation; blue represents a negative correlation. * means significantly different at the 0.05 level.

There was a significant positive correlation observed between oat yield, protein, and its component contents, β-GC, DMA, GNA, PNA, NFUE, NAE, PrNTA, NTR, and PoNAA (*p* < 0.05) (Figure 11). NFP exhibited a negative correlation with DMA and PrNTA. PrNTC displayed a negative correlation with PRO, GLI, PoNAA, and PoNAC. PoNAC showed a negative correlation with ALB, PrNTA, PrNTC, and NTR (*p* < 0.05).

## 4. Discussion

### 4.1. Oat Yield and Dry Matter Accumulation in Response to Nitrogen Fertilization

Previous studies investigating nitrogen fertilizer application rates have demonstrated a consistent trend, wherein wheat dry matter and grain yield initially increase and subsequently decrease as nitrogen application increases [25]; due to variations in soil fertility, crop types, and environmental conditions, the optimal nitrogen application rate for each crop should be determined to maximize the economic and ecological benefits of N fertilizer. The dry matter accumulation of L oats grains was highest at 180 kg·ha^−1^ of N application, then decreased significantly when the N application was increased to 240 kg·ha^−1^. Our two-year experiment revealed that nitrogen application enhanced oat dry matter accumulation and promoted its distribution to nutrient organs. However, excessive nitrogen application triggered nutrient organ overgrowth and delayed ripening [26,27], resulting in the proportion of dry matter allocated to the grain decreasing.

Crop yields are contingent upon the efficient translocation and distribution of assimilates, as well as the accessibility of nitrogen fertilizers. Nitrogen fertilization enhance dry matter accumulation and grain protein content [28,29], thereby improving oat yield and quality [30]. The interaction between oat varieties and nitrogen fertilizer is notable, significantly affecting the yield and yield components of different oat varieties; the late-maturing variety of oat exhibited a higher yield compared to the early- and medium-maturing varieties [9]. Moreover, significant variations in yield and yield components were observed among different oat varieties cultivated in various regions [31]. The relationship between yield and the amount of N applied follows a parabolic pattern; upon reaching a certain threshold of N application, further increases in N application result in a decrease in yield. The application of excessive nitrogen fertilizer led to reduced nitrogen use efficiency and oat yield, increased production costs, and environmental pollution [6]. Selecting oat varieties with high nitrogen efficiency and determining the optimal nitrogen application rate are crucial in determining the appropriate planting areas of oats. In this study, L oat varieties exhibited a higher nitrogen tolerance than E and M varieties. Notably, the yield of L oats peaked under N4, surpassing the peak yields of E and M oats by 32.92% and 17.60%, respectively.

### 4.2. Nutritional Quality of Oat Grains

Oats exhibit substantial nutritional value, with their grain protein content ranging from 11% to 16% [32]. Oat protein accumulates continuously during grain development, directly benefiting from nitrogen application [33]. In this study, the protein content of oats initially rose and subsequently declined in response to increasing nitrogen application rates, indicating that the beneficial effect of nitrogen on oat protein content was constrained. Notably, excessive nitrogen application led to a significant decrease in protein content. The protein contents in oat varieties of different maturities were significantly different; L oats showed the advantage of high-protein varieties compared with other oats, with the highest recorded protein content being 14.15%. Danuta Leszczyńska et al. [34] studied the mixed sowing of cereals in Poland, revealing that the protein content of oats ranged from 11.1% to 13.7%. Consequently, both environmental factors and oat variety characteristics significantly influence protein content. Oat protein can be classified into four main categories: albumin, globulin, gliadin, and glutenin. Albumin and globulin are rich in essential amino acids crucial to human nutrition and thereby influence the overall nutritional quality of oats. The content of gliadin and glutenin is related to the quality of oat flour, determining the processing quality of oats [35]. The effect of nitrogen application on protein component contents varies [36]; Li et al. [37] found that nitrogen application exerted varying degrees of impact on the enhancement of individual protein components: a significant promotion was evident in globulin content, followed by gliadin and glutelin. Our experiment found a positive correlation between total protein and various protein components. Nitrogen application significantly increased the content of oat protein components, with the most significant increase in globulin content. The highest content of grain protein was achieved at a nitrogen application rate of 180 kg·ha^−1^, followed by gliadin and glutelin, which is consistent with the above research results.

β-Glucan is a dietary fiber and a biologically active natural polysaccharide [38] occurring in grains of oats, barley, and wheat. The content of β-Glucan is significantly influenced by crop genotype and environmental factors; increasing nitrogen fertilizer application in different regions can improve oat β-Glucan [33]. This is because the increase in nitrogen fertilizer application can strengthen the photosynthesis of plants and increase the content of β-glucan synthesis precursor—glucose, thus increasing the synthesis amount of β-glucan. But Jackson et al. [39] believed that oat and barley β-Glucan is not significantly affected by the amount of nitrogen applied, and changes may also be caused by differences in variety and the environment in which the variety operates. The range for β-glucan content in high-protein oat varieties is 3.2–5.4% [36]. In this experiment, the highest record reached 4.92% in L oat grains, β-glucan increased to varying degrees compared to CK, and oat varieties and nitrogen led to significant differences in nutritional composition. Grain protein and β-glucan are independent indicators of grain nutritional quality and do not antagonize each other [36]. Therefore, high-protein oats rich in β-glucan are more highly favored [40].

### 4.3. Nitrogen Utilization and Transport Characteristics of Oats

The accumulation and transportation of nitrogen are closely related to the formation of grain yield. Reasonable nitrogen application helps to improve nitrogen accumulation and transportation, laying the foundation for high-quality and high-yield crops [41]. Varietal differences and N application rates both have a significant impact on the nitrogen content of cereals. N application increased the accumulation of nitrogen in the aboveground parts of cereals [42], but an excessive increase in N application reduced the nitrogen accumulation in various organs [43]. The nitrogen content in oat stems and leaves shows a declining trend over time, while in spikes and grains, it exhibits an increasing trend. We also found that the nitrogen accumulation in reproductive organs (spike + chaff, grain) was greater than that in nutrient organs (stem, leaf), and the nitrogen accumulation in N application treatments was significantly higher than that in CK. The peak nitrogen accumulation in L oats was observed with the N4 treatment, suggesting that exceeding a specific nitrogen application threshold can constrain the crop’s ability to absorb and accumulate nitrogen.

The nitrogen transport capacity before flowering reflects the nitrogen demand of the sink organ (grain) from the source organ (stem, leaf) and is closely related to the source–sink relationship [44]. The pre-flowering nitrogen translocation varies among different grain varieties, with nitrogen application enhancing this process in grains. Nitrogen accumulation during the flowering phase enhances the contribution of nitrogen from pre-flowering nutrient organs to grain partitioning, ultimately influencing the grain yield during the grouting period [45]. A heightened nitrogen transport capacity, however, correlates with diminished nutrient levels in the stem and leaves, subsequently elevating the likelihood of premature leaf senescence and plant lodging during the later crop growth phases. This, in turn, adversely impacts grain yield formation in the subsequent stages [46]. Excessive nitrogen application, however, significantly decreases the efficiency of nitrogen translocation to grains post-flowering, as evidenced by a notable decline in both the pre-flowering translocation and post-flowering accumulation amounts in oats. Furthermore, the post-flowering nitrogen accumulation and contribution in oats were found to be significantly lower compared to the pre-flowering nitrogen transport and contribution. Our findings underscore the significance of nitrogen in nutrient organs and highlight the primary contribution of pre-flowering nitrogen translocation to the grain nitrogen content. Optimizing nitrogen application is crucial for maximizing the nitrogen translocation efficiency from nutrient organs to reproductive organs and particularly enhancing pre-flowering nitrogen translocation to increase the grain nitrogen content, thereby improving nitrogen use efficiency in oats and ultimately enhancing their quality and yield.

The efficiency of nitrogen fertilizer utilization is primarily governed by three factors: crop nitrogen uptake, soil and fertilizer nitrogen availability, and nitrogen losses within the soil–plant system [47]. Notably, the quantity of nitrogen fertilizer applied is a crucial determinant of utilization efficiency. An increase in nitrogen application levels will lead to a decline in nitrogen fertilizer utilization efficiency [48]. Optimizing nitrogen fertilizer application ensures an adequate nutrient supply for crop growth and development while minimizing nitrogen loss and enhancing the fertilizer utilization efficiency [49]. In this study, as the nitrogen application rate increased, both nitrogen fertilizer productivity and nitrogen agronomic efficiency exhibited a notable decrease. As the nitrogen application rate for oats varied from 0 to 90 kg·ha^−1^, the nitrogen fertilizer use efficiency showed a significant increase trend, peaking at the N2 level; thereafter, NFUE demonstrated a pronounced decline. These findings suggest that an optimal nitrogen application rate is conducive to enhancing the nitrogen fertilizer utilization efficiency. Further research needs to be conducted, incorporating multi-site trials in other areas of the Loess Plateau.

## 5. Conclusions

Through a comparison of three oat varieties with different growth periods, it was discovered that nitrogen application favored oat growth, but excessive nitrogen application could inhibit it. The late-maturing oat variety achieved the highest yield (2946.30 kg·ha^−1^) and protein yield (14.15%) at a nitrogen application rate of 180 kg·ha^−1^. At 120 kg·ha^−1^, the late-maturing oat variety exhibited the highest β-glucan content of 4.92%. The nitrogen fertilizer use efficiency (NFUE) of all oat varieties was highest at 90 kg·ha^−1^, and the pre-flowering nitrogen translocation contribution (PrNTC) was higher than the post-flowering nitrogen accumulation contribution (PoNAC). There were significant positive correlations between oat yield, quality, and nitrogen accumulation and utilization. In summary, when nitrogen application rates ranged from 90 to 180 kg·ha^−1^, oats performed well in terms of yield, quality, and nitrogen utilization. The late-maturing oat variety is more suitable for planting in dryland regions of the Loess Plateau.

## Figures and Tables

**Table 1:** Soil characteristics before sowing in 2022 and 2023.

Year	Organic Matter	Total Soil Nitrogen	Alkali Hydrolyzed Nitrogen	Available Phosphorus	Available Potassium	pH
	g·kg^−1^	g·kg^−1^	mg·kg^−1^	mg·kg^−1^	mg·kg^−1^	
2022	14.20	1.04	80.44	12.70	181.86	7.6
2023	16.80	1.12	104.57	15.50	185.84	7.9

**Table 2:** Phenological period of different oat varieties in 2022 and 2023.

Year	Variety	Sowing	Seeding	Tillering	Jointing	Heading	Flowering	Maturity	Growth Duration/d
2022	E	7 April	14 April	24 April	4 May	20 May	8 June	3 July	80
	M	7 April	18 April	1 May	15 May	2 June	22 June	21 July	94
	L	7 April	22 April	6 May	21 May	9 June	2 July	8 August	108
2023	E	9 April	17 April	27 April	9 May	27 May	11 June	8 July	82
	M	9 April	20 April	5 May	19 May	8 June	27 June	25 July	96
	L	9 April	23 April	9 May	25 May	11 June	1 July	6 August	105

**Table 3:** Oat nitrogen fertilizer use efficiency-related indicators over the 2-year growing season.

Variety	N Application Rate	2022			2023		
		NFUE (%)	NFP (kg·kg^−1^)	NAE (kg·kg^−1^)	NFUE (%)	NFP (kg·kg^−1^)	NAE (kg·kg^−1^)
E	CK	/	/	/	/	/	/
	N1	45.18ab	32.96a	11.77a	28.75b	32.21a	6.63a
	N2	50.94a	22.52b	8.39b	39.78a	22.89b	5.84a
	N3	41.21b	17.39c	6.80c	36.30a	18.47c	5.68a
	N4	/	/	/	19.82c	11.49d	2.96b
	N5	/	/	/	9.54d	8.24f	1.85c
M	CK	/	/	/	/	/	/
	N1	36.74b	31.99a	11.40a	24.28b	34.43a	5.84a
	N2	56.56a	23.71b	9.98b	40.55a	24.37b	5.31ab
	N3	51.54ab	18.19c	7.90c	39.32a	18.88c	4.58b
	N4	/	/	/	27.71b	13.92d	4.39b
	N5	/	/	/	10.78c	10.12f	2.97c
L	CK	/	/	/	/	/	/
	N1	38.01b	32.33a	11.07a	34.66b	40.71a	7.23a
	N2	69.19a	24.89b	10.71ab	57.38a	29.16b	6.84ab
	N3	59.35a	19.83c	9.20b	49.38a	23.10c	6.36ab
	N4	/	/	/	47.32a	16.37d	5.21b
	N5	/	/	/	29.15b	11.13f	2.76c
F-value	Variety (V)	*	***	**	***	***	***
	Nitrogen (N)	***	***	***	***	***	***
	V × N	*	**	**	*	***	*

Note: Different lowercase letters for the same variety indicate that the difference between different nitrogen fertilizer levels of the same variety is significant at the *p* < 0.05 level; * indicates that the difference is significant at the 0.05 level; ** indicates that the difference is significant at the 0.01 level; *** indicates that the difference is significant at the 0.001 level; and ns indicates that the difference is not significant. The same is true for the following Table 4.

**Table 4:** Oat nitrogen translocation efficiency-related indicators over the 2-year growing season.

Variety	N Application Rate	2022					2023				
		PrNTA (kg·ha^−1^)	PrNTC (%)	NTR (%)	PoNAA (kg·ha^−1^)	PoNAC (%)	PrNTA (kg·ha^−1^)	PrNTC (%)	NTR (%)	PoNAA (kg·ha^−1^)	PoNAC (%)
E	CK	26.56c	55.11c	52.85c	21.69c	44.89a	27.12d	55.87b	49.02d	21.41b	44.13a
	N1	41.82b	58.75b	59.94ab	29.38b	41.25b	37.18c	60.23ab	53.56bc	24.62ab	39.77ab
	N2	52.52a	59.09b	64.39a	36.35a	40.91b	48.15b	62.61ab	57.43a	28.77ab	37.39ab
	N3	53.58a	64.20a	58.57b	29.90b	35.80c	52.32a	63.53a	57.89a	30.06a	36.47a
	N4	/	/	/	/	/	47.06b	62.37ab	56.15ab	28.70ab	37.63ab
	N5	/	/	/	/	/	37.27c	58.53ab	51.00cd	26.64ab	41.47ab
M	CK	29.45d	59.32b	53.50d	19.96b	40.68a	28.22e	63.36b	46.54d	16.32b	36.64a
	N1	41.04c	62.40ab	56.86b	24.72b	37.60ab	36.61d	64.18b	51.35c	20.40ab	35.82a
	N2	56.32b	63.30ab	60.50c	31.97a	36.70ab	54.17b	72.29a	58.57ab	20.95ab	27.71b
	N3	67.30a	67.92a	64.15a	32.66a	32.08b	63.05a	74.56a	61.32a	21.50a	25.44b
	N4	/	/	/	/	/	63.70a	73.85a	60.98a	22.55a	26.15a
	N5	/	/	/	/	/	47.19c	70.68a	56.44b	19.54ab	29.32b
L	CK	35.75c	54.16a	53.28b	30.26b	45.84a	31.72d	51.20b	46.59c	30.01b	48.80a
	N1	46.79b	55.19a	57.03ab	38.02b	44.81a	43.06c	54.06ab	52.28b	26.94b	45.94ab
	N2	65.55a	55.66a	61.26a	50.99a	44.34a	61.64b	56.21ab	60.34a	47.91a	43.79ab
	N3	72.62a	58.67a	61.66a	52.60a	41.33a	65.46b	56.77ab	60.89a	50.24a	43.23ab
	N4	/	/	/	/	/	78.66a	60.65a	59.22a	51.02a	39.35b
	N5	/	/	/	/	/	69.88ab	58.83ab	58.51a	48.99a	41.17ab
F-value	Variety (V)	***	***	ns	***	***	***	***	*	***	***
	Nitrogen (N)	***	***	***	***	***	***	***	***	***	***
	V × N	*	ns	*	*	ns	***	ns	*	*	ns

## Data Availability

Data are contained within the article.
